# Epitaxial Relationships between Calcium Carbonate and Inorganic Substrates

**DOI:** 10.3390/ijms150916320

**Published:** 2014-09-15

**Authors:** Taewook Yang, Jae Young Jho, Il Won Kim

**Affiliations:** 1 School of Chemical and Biological Engineering, Seoul National University, Seoul 151-742, Korea; E-Mail: taugi1@snu.ac.kr; 2 Department of Chemical Engineering, Soongsil University, Seoul 156-743, Korea

**Keywords:** biomineralization, polymorphism, calcium carbonate, heterogeneous nucleation, epitaxy

## Abstract

The polymorph-selective crystallization of calcium carbonate has been studied in terms of epitaxial relationship between the inorganic substrates and the aragonite/calcite polymorphs with implication in bioinspired mineralization. EpiCalc software was employed to assess the previously published experimental results on two different groups of inorganic substrates: aragonitic carbonate crystals (SrCO_3_, PbCO_3_, and BaCO_3_) and a hexagonal crystal family (α-Al_2_O_3_, α-SiO_2_, and LiNbO_3_). The maximum size of the overlayer (aragonite or calcite) was calculated for each substrate based on a threshold value of the dimensionless potential to estimate the relative nucleation preference of the polymorphs of calcium carbonate. The results were in good agreement with previous experimental observations, although stereochemical effects between the overlayer and substrate should be separately considered when existed. In assessing the polymorph-selective nucleation, the current method appeared to provide a better tool than the oversimplified mismatch parameters without invoking time-consuming molecular simulation\.

## 1. Introduction

Biological mineralization has been of great interest because of the intricate structures observed in hierarchical levels, which is uncommon in synthetic inorganic systems [1]. The mollusk shells (calcium carbonate) are probably the most extensively studied systems, and their nacre structures have been investigated in depth to reveal diverse phenomena of non-classical crystallization. Some of the interesting observations include the layered micro-crystals with tightly controlled orientation, the presence of nanoparticles as the constituting domains of the micro-crystals, and the formation of metastable polymorph (aragonite) [1,2,3,4].

The most thermodynamically stable polymorph of calcium carbonate is calcite under ambient conditions, and the metastable aragonite formation is very often observed in mollusk shells [1,2]. Although the stabilization of aragonite is possible in the presence of magnesium or at elevated temperature in the synthetic systems [5], it has become clear that the phenomenon in biological systems is intimately related to the interfacial phenomenon between the biomacromolecules and the crystals of calcium carbonate [2,4,6,7].

The bioinspired crystallization to mimic the polymorph selection at the biological interfaces has been studied with various substrates. Studies involving organic substrates, such as biomacromolecules and self-assembled monolayers, suggested the importance of the geometric and stereochemical match for the control of polymorphs and orientations of crystals [6,8,9]. Systematic studies on inorganic substrates also suggested that close epitaxy could be the key factor to form the metastable polymorph of aragonite, although the stereochemical effect could be an additional factor that increased the selectivity [10,11].

In the present study, we explored a simple method to assess the polymorphs-selective nucleation of aragonite. The purpose was to establish a method that could help to rapidly screen the nucleating substrates before further time-consuming experimental investigations, and the method could be also helpful to better understand the experimentally observed phenomena. Six inorganic substrates from two previous publications were examined to construct and verify the method. They were SrCO_3_, PbCO_3_, BaCO_3_, α-Al_2_O_3_, α-SiO_2_, and LiNbO_3_ [10,11]. The attempted method employed a computational program (EpiCalc) that could examine the phase coherence of two different crystal planes [12,13]. (Although the EpiCalc software has been used in many examples to explain the oriented crystallization, it has not been extensively utilized to assess the polymorph-selective crystallization [12,13,14,15,16]). The epitaxial matches of aragonite and calcite with the substrates were compared to predict the relative preference of CaCO_3_-polymorph nucleation. The strategy employed in the present work was able to calculate the results that matched the experimental observations on these substrates, although the restriction related to the stereochemistry was also revealed.

## 2. Results and Discussion

**Figure 1:**
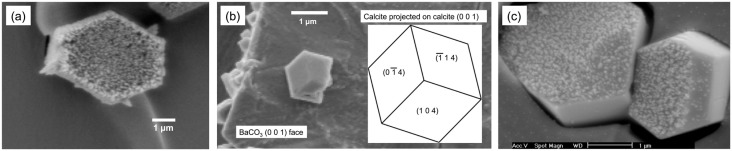
Scanning electron microscopy (SEM) images of (**a**) aragonite needles nucleated on SrCO_3_ (shown perpendicular to the (0 0 1) plane); (**b**) calcite formed on BaCO_3_ (shown perpendicular to the (0 0 1) plane) along with the simulated morphology of calcite projected on the (0 0 1) plane of calcite; and (**c**) nano-domains of calcium carbonate nucleated on SrCO_3_ [17].

The nucleation behavior of the inorganic compounds shown in Table 1 could be summarized as follows according to the previous publications [10,11]. First, SrCO_3_ possessed the closest unit cell parameters to aragonite among the carbonate crystals with aragonitic crystal structure, and it nucleated aragonite exclusively under the crystallization conditions that favored calcite formation at 4–6 °C. PbCO_3_ showed predominant formation of aragonite with some calcite under the same conditions. BaCO_3_, of which unit cell parameters were farthest from those of aragonite, could not nucleate aragonite, and only calcite crystals were observed. In addition, the orientations of the aragonite and calcite crystals with respect to the substrates followed the stereochemical relationships between carbonates, *i.e.*, the (0 0 1) planes of substrates and growing crystals were parallel (Figure 1a,b) [10,17]. Note that the crystal orientations of calcium carbonate governed by the stereochemistry of carbonate have been often observed on various surfaces [9,18,19]. Also, the early nucleation-and-growth domains as small as 20 nm had been observed on SrCO_3_, which clearly showed the populated (0 0 1) faces of SrCO_3_ (Figure 1c) [17].

**Table 1:** Crystal structures of the inorganic compounds in the present study [20,21,22,23,24,25].

Crystals	Space Group	*a* (Å)	*b* (Å)	*c* (Å)	α (°)	β (°)	γ (°)
Aragonite	*Pmcn*	4.961	7.967	5.740	90	90	90
SrCO_3_	*Pmcn*	5.090	8.358	5.997			
PbCO_3_	*Pmcn*	5.179	8.492	6.141			
BaCO_3_	*Pmcn*	5.313	8.896	6.428			
Calcite	*R*–3*c*	4.990	-	17.06	90	90	120
*α*-Al_2_O_3_	*R*–3*c*	4.760	-	12.99			
*α*-SiO_2_	*P*3_1_21	4.916	-	5.405			
LiNbO_3_	*R*–3*c*	5.212	-	14.36			

Second, the crystal substrates, which belong to the trigonal space group, were carefully selected by Pokroy and Zolotoyabko to study the effects of lattice match without the contribution of stereochemistry [11]. In addition, the (0 0 1) surface (in the hexagonal setting) was selected to have similar arrangements of cations with those of calcium ions in the (0 0 1) planes of aragonite and calcite. The authors observed that the crystallization performed at about 22 °C formed aragonite on all substrates, although the predominantly generated crystals were calcite in all cases. The crystal orientations of both aragonite and calcite were such that their (0 0 1) planes were parallel to the (0 0 1) surfaces of the substrates, which further supported the heterogeneous nucleation through epitaxy.

The previously described systems were chosen for the current study because the inorganic substrates of rigid nature presented a better opportunity for the epitaxy analysis than organic substrates of which lattice parameters could be more easily altered at the interfaces. We also note here that the comparative analysis was limited within each system: aragonitic carbonate crystals (SrCO_3_, PbCO_3_, and BaCO_3_) and a hexagonal crystal family (α-Al_2_O_3_, α-SiO_2_, and LiNbO_3_).

The calculations based on EpiCalc required the cell parameters from the two-dimensional repeating structures of the planes of interest. Since the planes of interest were (0 0 1), the cell parameters were simply {*a*, *a*, γ} of the unit cell parameters of calcite and {*a*, *b*, γ} for the rest of the inorganic compounds (Table 1). While the detailed description on the EpiCalc calculations can be found in the original paper [12], a concise summary is as follows. EpiCalc examines the lattice coherence between overlayer and substrate, assuming that their potential energy surfaces correspond to their lattice periodicities, which can be described as simple plane waves [12,13]. Also, it can specify a range of azimuthal angle and the numbers of overlayer unit cells for the calculation. The calculation yields a dimensionless potential (*V*/*V*_o_) that indicates the degree of commensurability [12]. For the sufficiently large overlayer size, when all lattice points of the overlayer matches with the substrate lattice points (commensurate epitaxy), the *V*/*V*_o_ value is 0. The *V*/*V*_o_ value is 0.5, when the supercell vertices of the overlayer matches with the substrate lattice points (coincident epitaxy). The *V*/*V*_o_ value 1 means incommensurate. In the overlayer/substrate combinations in the present study, commensurate epitaxy is absent, and our purpose is to calculate the maximum overlayer size of aragonite and calcite for each substrate that generate *V*/*V*_o_ value less than 0.5 to assess the relative propensity of the polymorph-selective heterogeneous nucleation.

**Figure 2:**
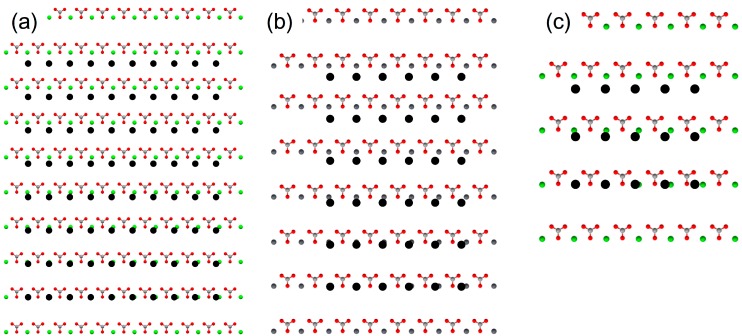
Molecular models to show the maximum supercell dimensions of aragonite (0 0 1) that generated the dimensionless potential (*V*/*V*_o_) less than 0.5 with the (0 0 1) planes of the substrates: (**a**) SrCO_3_; (**b**) PbCO_3_; and (**c**) BaCO_3_. The filled black circles are the lattice points of aragonite overlayer.

The results of EpiCalc calculations are summarized in Table 2. Among the substrates of the same orthorhombic crystal structures as aragonite, the supercell dimensions that generated the *V*/*V*_o_ less than 0.5 were in the order SrCO_3_ > PbCO_3_ > BaCO_3_ as expected since the unit cell parameters, therefore the two-dimensional cell parameters, deviated farther as the size of cation increased. They were depicted in Figure 2. The supercell dimensions of 9 × 7 (on SrCO_3_), 5 × 5 (on PbCO_3_), and 4 × 2 (on BaCO_3_) corresponded to ca. 4.5 nm × 5.6 nm (on SrCO_3_), 2.5 nm × 4.0 nm (on PbCO_3_), and 2.0 nm × 1.6 nm (on BaCO_3_), respectively. This combined with the experimental results from the previous publications indicated that the critical nuclei size to form aragonite could be about 2–3 nm for this family of substrates, which is in the range recently reported [26]. Interestingly, calcite (0 0 1) did not show any epitaxial match; even the 1 × 1 cell generated the *V*/*V*_o_ more than 0.5. This suggested that the sparsely populating calcite crystals on BaCO_3_ could be from the attached growth of the homogeneously nucleated calcite crystals supported by the stereochemical match of carbonate groups. Note that the oriented attachment of nanocrystals has been observed in calcium carbonate as well as other systems, such as iron oxyhydroxide and calcium oxide [27,28,29]. Overall, the EpiCalc calculations were successful in assessing the preferential nucleation of aragonite on SrCO_3_ and PbCO_3_, although the contribution of stereochemical effects is yet to be quantified.

We note here that the lack of the consideration on the well-known stereochemical effects is the major limitation of the assessment using EpiCalc. When stereochemical effects of carbonate orientations were considered as previously shown, the epitaxial analysis between calcite and the (0 0 1) surfaces of aragonitic substrates was restricted to the (0 0 1) plane of calcite. If the stereochemical restraint is not considered, only lattice periodicities are assessed by EpiCalc. Then, unrealistic epitaxial relationships could be obtained with other crystallographic planes of calcite, as explained in the next paragraph.

**Table 2:** The maximum size of supercell dimensions along with the azimuthal angles that generated the dimensionless potential (*V*/*V*_o_) less than 0.5, when the epitaxy relationships between the (0 0 1) planes of the various substrates and the overlayers (aragonite and calcite) were calculated.

Substrate	Aragonite			Calcite		
	Supercell Dimensions	*V*/*V*_o_	Angle (°)	Supercell Dimensions	*V*/*V*_o_	Angle (°)
SrCO_3_	9 × 7	0.491	0	No match *		
PbCO_3_	5 × 5	0.458	0	No match		
BaCO_3_	4 × 2	0.433	0	No match		
Al_2_O_3_	6 × 5	0.473	60	6 × 6	0.423	0
SiO_2_	10 × 10	0.487	60	21 × 21	0.463	0
LiNbO_3_	4 × 3	0.489	60	12 × 12	0.438	0

* No match: *V*/*V*_o_ over 0.5 for the 1 × 1 cell.

**Figure 3:**
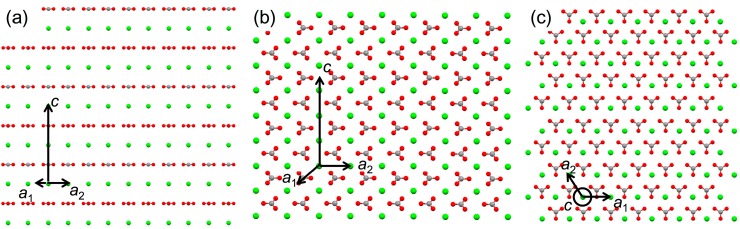
Molecular models of calcite (1 0 0) (**a**); (1 0 4) (**b**); and (0 0 1) (**c**) planes to show the different orientations of carbonate ions.

Among the frequently observed surfaces of calcite [30], highly common (1 0 4) as well as some low index surfaces, such as (1 0 0) and (0 1 2), were examined. The results concerning the overlayers of calcite (1 0 0) and (1 0 4) are displayed in Table 3. (The (0 1 2) did not generate a good match.). The cell parameters from the two-dimensional repeating structures for the overlayers were as follows: {*b*_1_ = 4.990 Å, *b*_2_ = 17.06 Å, β = 90°} for calcite (1 0 0); {*b*_1_ = 4.990 Å, *b*_2_ = 8.096 Å, β = 90°} for calcite (1 0 4). For example, the 10 × 9 supercell of calcite (1 0 0) on SrCO_3_ corresponded to *ca.* 5.0 nm × 15.4 nm, which appeared to suggest the calcite formation on SrCO_3_ although no calcite formation was experimentally observed [10]. Again, the impractical results were obtained because the stereochemical effects of carbonate orientations were ignored. Figure 3 shows the different orientations of carbonate ions in the (1 0 0), (1 0 4), and (0 0 1), the last being the same orientation as the (0 0 1) surfaces of the aragonitic substrates. Altogether, the current analysis shows that the evaluation using EpiCalc should be cautiously inspected when the stereochemical effects, which cannot be neglected, are suspected.

**Table 3:** The maximum size of supercell dimensions along with the azimuthal angles that generated the dimensionless potential (*V*/*V*_o_) less than 0.5, when the epitaxy relationships between the (0 0 1) planes of the substrates and the (1 0 0) or (1 0 4) planes of calcite were calculated.

Substrate	Calcite (1 0 0)			Calcite (1 0 4)		
	Supercell Dimensions	*V*/*V*_o_	Angle (°)	Supercell Dimensions	*V*/*V*_o_	Angle (°)
SrCO_3_	10 × 9	0.499	0	11 × 11	0.482	0
PbCO_3_	12 × 12	0.493	0	7 × 6	0.463	0
BaCO_3_	4 × 3	0.439	0	4 × 3	0.475	0

**Figure 4:**
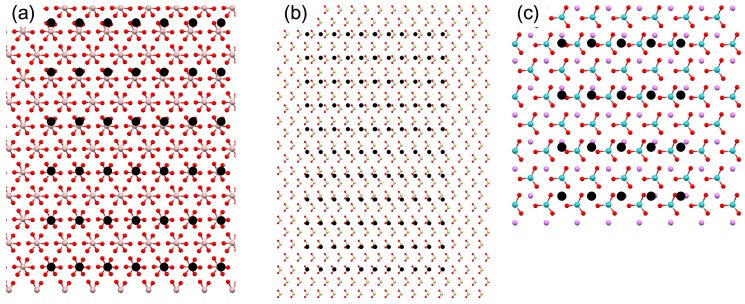
Molecular models to show the maximum supercell dimensions of aragonite (0 0 1) that generated the dimensionless potential (*V*/*V*_o_) less than 0.5 with the (0 0 1) planes of the substrates: (**a**) Al_2_O_3_; (**b**) SiO_2_; and (**c**) LiNbO_3_. The filled black circles are the lattice points of aragonite overlayer.

**Figure 5:**
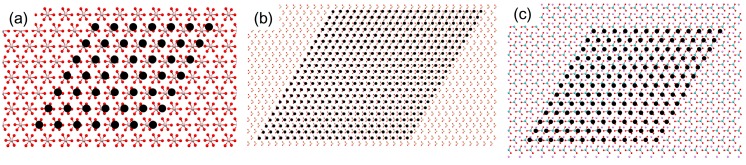
Molecular models to show the maximum supercell dimensions of calcite (0 0 1) that generated the dimensionless potential (*V*/*V*_o_) less than 0.5 with the (0 0 1) planes of the substrates: (**a**) Al_2_O_3_; (**b**) SiO_2_; and (**c**) LiNbO_3_. The filled black circles are the lattice points of calcite overlayer.

Further calculations for the Al_2_O_3_, SiO_2_, and LiNbO_3_ confirmed the utility of EpiCalc in assessing the polymorph-selective nucleation when the stereochemical influence was absent (Table 2). The cases of aragonite (0 0 1) overlayer are shown in Figure 4. The supercell dimensions of 6 × 5 (on Al_2_O_3_), 10 × 10 (on SiO_2_), and 4 × 3 (on LiNbO_3_) corresponded to *ca.* 3.0 nm × 4.0 nm (on Al_2_O_3_), 5.0 nm × 8.0 nm (on SiO_2_), and 2.0 nm × 2.4 nm (on LiNbO_3_), respectively. The cases of calcite (0 0 1) overlayer were shown in Figure 5. The supercell dimensions of 6 × 6 (on Al_2_O_3_), 21 × 21 (on SiO_2_), and 12 × 12 (on LiNbO_3_) corresponded to *ca.* 3.0 nm × 3.0 nm (on Al_2_O_3_), 10.5 nm × 10.5 nm (on SiO_2_), and 6.0 nm × 6.0 nm (on LiNbO_3_) for the parallelogram with the acute angle 60°, respectively. Comparative assessment of aragonite (0 0 1) and calcite (0 0 1) indicated predominant calcite formation on SiO_2_ and LiNbO_3_ and comparable formation on Al_2_O_3_. These results were in good agreement with the previous experimental observation, where the predominant calcite formation along with some aragonite was detected in all cases [11].

**Figure 6:**
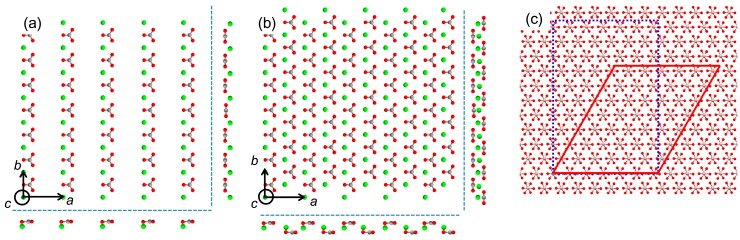
Molecular models of the (0 0 1) plane of aragonite along with the side views (outside dashed lines): (**a**) orthorhombic and (**b**) pseudo-hexagonal arrangements; (**c**) Maximum supercell dimensions of aragonite (0 0 1) that generated the dimensionless potential (*V*/*V*_o_) less than 0.5 with the (0 0 1) plane of Al_2_O_3_: orthorhombic (dotted line) *vs.* pseudo-hexagonal (solid line) arrangements.

We also note here that invoking pseudo-hexagonal argument of orthorhombic aragonite (Figure 6a) as seen in the previous publication is not necessary for the EpiCalc calculations [11]. The previous publication utilized simple mismatch parameters, and it was forced to construct pseudo-hexagonal structure from the orthorhombic aragonite. This was to match the substrate symmetry since the analysis of mismatch parameters was not feasible when the unit cells of substrate and overlayer were of different symmetry. The pseudo-hexagonal structure is shown in Figure 6b. The arrangement of carbonate ions, as shown in the side views of Figure 6b, clearly shows that the pseudo-hexagonal structure is oversimplification. In fact, the alternating rows of calcium ions in *b* direction are about 0.1 Å apart in *c* direction as well. Figure 6c shows the representative comparison between the calculation results using orthorhombic and pseudo-hexagonal arrangements of aragonite on Al_2_O_3_. The supercell dimension of aragonite (0 0 1) overlayer in the orthorhombic arrangement was 6 × 5 corresponding to *ca.* 3.0 nm × 4.0 nm. That of aragonite (0 0 1) overlayer in the pseudo-hexagonal arrangement (*b*_1_ = 4.961 Å, *b*_2_ = 4.694 Å, β = 121.9°) was 6 × 7 corresponding to *ca.* 3.0 nm × 3.3 nm for the parallelogram with the acute angle 58.1°. Overall, the EpiCalc calculation was effective in determining the phase coherence of layers in different two-dimensional symmetries without the imprecise pseudo-hexagonal assumption for aragonite.

## 3. Experimental Section

Two anhydrous polymorphs of calcium carbonate (CaCO_3_) examined in the present work were aragonite and calcite [20,21]. The inorganic substrates, from which the crystals of calcium carbonate were nucleated in the previous publications [10,11], could be categorized into two groups (Table 1). The first group is of the same crystal structure of orthorhombic aragonite (*Pmcn*), except that the cations were replaced with strontium, lead, and barium to form SrCO_3_, PbCO_3_, and BaCO_3_, respectively [21,25]. The other group is of hexagonal crystal family, where their (0 0 1) planes contain a trigonal symmetry of cations: sapphire (α-Al_2_O_3_), trigonal quartz (α-SiO_2_), and lithium niobate (LiNbO_3_) [22,23,24].

The crystal structures of the two anhydrous CaCO_3_ polymorphs as well as all the inorganic substrates were taken from the American Mineralogist Crystal Structure Database [31]. To analyze their lattice periodicities and atomic arrangements, Mercury (version 3.1; The Cambridge Crystallographic Data Centre, Cambridge, UK) software was utilized [32]. When the atomic arrangements were shown, each element was color-coded (Li: purple; C: gray; O: red; Al: pink; Si: light yellow; Ca: light green; Sr: green; Nb: light blue; Ba: dark green; Pb: dark gray).

EpiCalc (version 5.0) software was employed to assess the degree of lattice match between the inorganic substrates and the two polymorphs of calcium carbonate (aragonite and calcite) [33]. The lattice periodicities of the substrate and the overlayer were set with cell parameters {*a*_1_, *a*_2_, and α} and {*b*_1_, *b*_2_, and β}, respectively, through the analysis of the crystal planes of interest using Mercury. Then, their phase coherence was quantified throughout the varied azimuthal angle (θ) between 0° and 180° in the interval of 0.01° [12,13]. The quantification was measured through a dimensionless potential (*V*/*V*_o_) that ranges from 0 and 1 indicating a closer geometric match with a smaller value.

Our goal was to use the EpiCalc as a screening tool to quickly assess the possibility of nucleating the metastable polymorph of aragonite. Therefore, aragonite and calcite were compared as overlayers in terms of the closeness to the individual substrate using the following strategy. For each pair of the substrate and the overlayer, the calculation was performed with varying size of the overlayer supercell. The variation was initially with N × N supercells, from 25 × 25 all the way down to 1 × 1 (the two dimensional repeating unit). After finding the largest supercell (e.g., M × M) giving the *V*/*V*_o_ below 0.5, which usually indicated coincident epitaxy, the supercell was enlarged to (M + 3) × M, (M + 2) × M, (M + 1) × M, M × (M + 1), M × (M + 2), and M × (M + 3) for more precise evaluation.

## 4. Conclusions

In summary, the polymorph-selective nucleation of calcium carbonate was assessed using the software EpiCalc that could examine the epitaxial relationship between the nucleating substrates and the overlayers of calcium carbonate crystals. Also, the reviewed cases of inorganic substrates offered a unique opportunity to examine the utility of EpiCalc in the polymorph selection because of the rigid characteristics of the inorganic crystals and the systematic nature of the cases. For the trigonal substrates where the stereochemical effects did not exist, the calculations suggested the mixed nucleation of aragonite and calcite, the latter being in predominant or comparable quantities, which was in good agreement with the experimental observations of the previous publication [11]. For the aragonitic substrates, the calculations were also in good agreement with the experimental results [10], only if the stereochemistry of carbonate was accounted in advance. Further studies on the quantitative analysis of the stereochemical effects would be necessary to improve the current method. Overall, the current results indicated that our strategy could serve as a useful method to estimate the polymorph-selective nucleation of calcium carbonate, and it could be probably extended to different systems.
